# Habitat selection during ungulate dispersal and exploratory movement at broad and fine scale with implications for conservation management

**DOI:** 10.1186/s40462-014-0015-4

**Published:** 2014-07-26

**Authors:** Joshua Killeen, Henrik Thurfjell, Simone Ciuti, Dale Paton, Marco Musiani, Mark S Boyce

**Affiliations:** Department of Biological Sciences, University of Alberta, Edmonton, AB T6G 2E9 Canada; Marine Evolution and Conservation Group, Centre of Evolutionary and Ecological Studies, University of Groningen, PO Box 11103 CC, Groningen, The Netherlands; Department of Biometry and Environmental System Analysis, University of Freiburg, Freiburg, 79106 Germany; Faculty of Environmental Design, University of Calgary, Calgary, AB T2N 1 N4 Canada

**Keywords:** Alberta, *Cervus elaphus*, Dispersal, Elk, Habitat selection, Migration, Step selection functions

## Abstract

**Background:**

Dispersal has a critical influence on demography and gene flow and as such maintaining connectivity between populations is an essential element of modern conservation. Advances in satellite radiotelemetry are providing new opportunities to document dispersal, which previously has been difficult to study. This type of data also can be used as an empirical basis for defining landscapes in terms of resistance surfaces, enabling habitat corridors to be identified. However, despite the scale-dependent nature of habitat selection few studies have investigated selection specifically during dispersal. Here we investigate habitat selection during and around dispersal periods as well as the influence of age and sex on dispersal for a large ungulate.

**Results:**

Of 158 elk (*Cervus elaphus*) tracked using GPS radiotelemetry almost all dispersers were males, with individuals dispersing up to 98 km. The dispersal period was distinct, with higher movement rates than before or after dispersal. At fine scale elk avoided the most rugged terrain in all time periods, but to a greater extent during and after dispersal, which we showed using step selection functions. In contrast, habitat selection by resident elk was less affected by ruggedness and more by an attraction to areas of higher forage availability. At the broad scale, however, movement corridors of dispersers were characterized by higher forage availability and slightly lower ruggedness then expected using correlated random walks.

**Conclusions:**

In one of the first examples of its kind we document complete long-distance dispersal events by an ungulate in detail. We find dispersal to be distinct in terms of movement rate and also find evidence that habitat selection during dispersal may differ from habitat selection in the home-range, with potential implications for the use of resistance surfaces to define conservation corridors.

**Electronic supplementary material:**

The online version of this article (doi:10.1186/s40462-014-0015-4) contains supplementary material, which is available to authorized users.

## Background

Dispersal is a fundamental process in ecology and evolution, affecting individual fitness as well as demography, genetic structure, and species distributions [1-4]. Dispersal is likewise of central importance for managing animal populations; maintaining connectivity among populations is considered to be an essential part of modern conservation [5]. Although theoretical work on dispersal is well developed, empirical studies are generally lacking because of the difficulties associated with observing and quantifying the dispersal process [6]. However, new advances in satellite radiotelemetry now permit opportunities to document dispersal that were previously unattainable [7].

Landscape connectivity describes how the movement of animals is linked to landscape structure [8]. The way in which movement among populations is affected by environmental conditions is important for predicting the effects of landscape modification and habitat fragmentation, and in prioritising which habitats to protect. The most widely used method for maintaining connectivity is the conservation corridor, a protected area of landscape that facilitates the movement of organisms between populations [5]. One approach has been to map resistance surfaces to characterize how environmental variables affect animal movement, and to use these surfaces to model connectivity and thus to inform management decisions relating to corridors [9]. As the use of Geographic Information Systems (GIS) has become more widespread, landscapes are increasingly defined using resistance surfaces, allowing the use of methods such as least-cost path analysis to help determine the most effective placement of corridors [10]. However, the effectiveness of such an approach is highly dependent on the accurate assignment of resistance values to landscape units, yet this has mostly been a subjective process [9,11]. Using empirical data generally leads to more robust and more readily justifiable conclusions [12].

Where empirical data have been used, it frequently has been assumed that habitat selection parameters derived from movement within home ranges can be extrapolated to movement during dispersal [13,14]. Corridors aim to promote connectivity between populations and therefore need to facilitate dispersal movement, making this assumption a potential source of error [9]. There is currently little information to suggest whether this assumption is reasonable or not, although Soulsbury et al. [15] found that red foxes (*Vulpes vulpes*) have unique patterns of habitat selection during dispersal, while Newby [16] found that cougars (*Puma concolor*) do not. The general lack of data on this subject probably reflects the difficulty of measuring and tracking dispersal [6]. Predicting which animals are likely to disperse can be difficult, so tracking those specific individuals is often not possible. Even with a large sample size the number of dispersing individuals within a population can be small [13,17]. Given that badly designed corridors run the risk of acting as population sinks or simply wasting financial resources and eroding the support of stakeholders [11,18], this is an important issue to address. Greater understanding of habitat selection by animals during dispersal should lead to better informed conservation management decisions.

Here we investigate how age and sex influence the likelihood of dispersal for an ungulate. Sex differences in juvenile dispersal are common and for mammals it is most often young males that disperse [19]. In a polygynous mating system males are expected to have more variation in reproductive success because a male must secure a territory or become dominant to increase reproductive success [20]. Therefore it is important for juvenile males to avoid sexual competition with older and more powerful males, and consequently dispersal is favoured [21]. We then investigate whether habitat selection during dispersal differs from habitat selection during home-ranging and other behaviours. We hypothesise that habitat selection during dispersal will have a unique pattern based on ease of movement through the landscape.

During 2007-2011 we monitored elk (*Cervus elaphus*) in the Rocky Mountains of North America using GPS telemetry to address these questions. Elk herds in this area may be migratory, in which animals move to higher altitudes in spring and summer to gain access to high-quality forage, or partially migratory, in which some animals remain resident in winter ranges throughout the year [22]. Dispersal occasionally takes place, when an animal leaves its home range and does not return. We expected that young male elk would be more likely to disperse than females [23]. Many male cervids are known to disperse from their natal home range, including elk [24-26]. We also predicted that dispersers would select habitats based more on ease of movement through the landscape than forage quality and we expected this pattern to be clear during dispersal, while before and after dispersal we expected forage quality to be more important in habitat selection. The dispersal period also was expected to be distinct in terms of movement parameters, with faster and more directional movement. Furthermore we expected there to be distinct movement behaviours at the fine-scale; shorter foraging movements and longer directional movements [27]. We predicted that longer steps would be selected based more on ease of movement while shorter steps would be selected based more on forage quality. We used resident individuals for further comparison, because of their consistent home-ranging behaviour, and we expected habitat selection to be consistently less affected by ease of movement and more affected by forage quality for these individuals.

We tested these predictions by first differentiating dispersal from other types of movement, such as migration and residency, on the basis of the measurement Net-Squared Displacement (NSD) [28] and extracting telemetry points within the dispersal period. We quantified the distance and duration of dispersal using these data. We then used step selection functions (SSF) and step-length analysis to compare fine-scale habitat selection between time periods for dispersing individuals and for matched resident individuals [29]. We also investigated the dispersal period itself using segmented regression to split movement behaviours into different scales of movement, before analysing each separately [30]. Finally, we used correlated random walks to analyse selection at a broad scale. While step selection functions can be used to analyse animal behavioural decisions during movement at a scale measured in hours, correlated random walks can be used to analyse movement at the scale of a complete dispersal pathway.

## Results

### Dispersal and movement parameters

Of the 132 elk (94 female, 38 male) with complete data, 97 were identified as migratory and 17 as resident. There were a total of 16 dispersal events, all but one undertaken by males, 9 of which completed dispersal and 7 of which were exploratory movements. Of the males tracked for the required time period (at least 1^st^ April to 31^st^ October), this translates to 39% of males dispersing (24% completing dispersal, 16% showing an exploratory movement) (Table 1).

**Table 1:** **Movement classifications using net squared displacement**

	**Male (** ***n*** **= 38)**	**Female (** ***n*** **= 94)**	**Total**	**Percentage (%)**
Migratory	26	71	97	73
Resident	2	15	17	13
Other/not classified	10	8	18	14
Dispersal event	15 (6)	1 (1)	16	

A total of 132 animals were observed between 2007 and 2011, with data from at least April 1^st^ to October 31^st^, using GPS radiotelemetry and were classified by movement type; migrant or resident. Some animals had an atypical movement pattern and were classified as other/not classified. The number of animals which had a dispersal period (number of those which were exploratory movements in brackets) was counted. Depending on the availability of data in the year following dispersal some dispersing animals could also be classified as migrants or residents. Those without data in the following year fall under the ‘other’ category. Classifications were made by classifying and inspecting Net Squared Displacement graphs *sensu* Mysterud et al. 2011 [44] (see Additional file 1: Figure S1).

**Figure 1:**
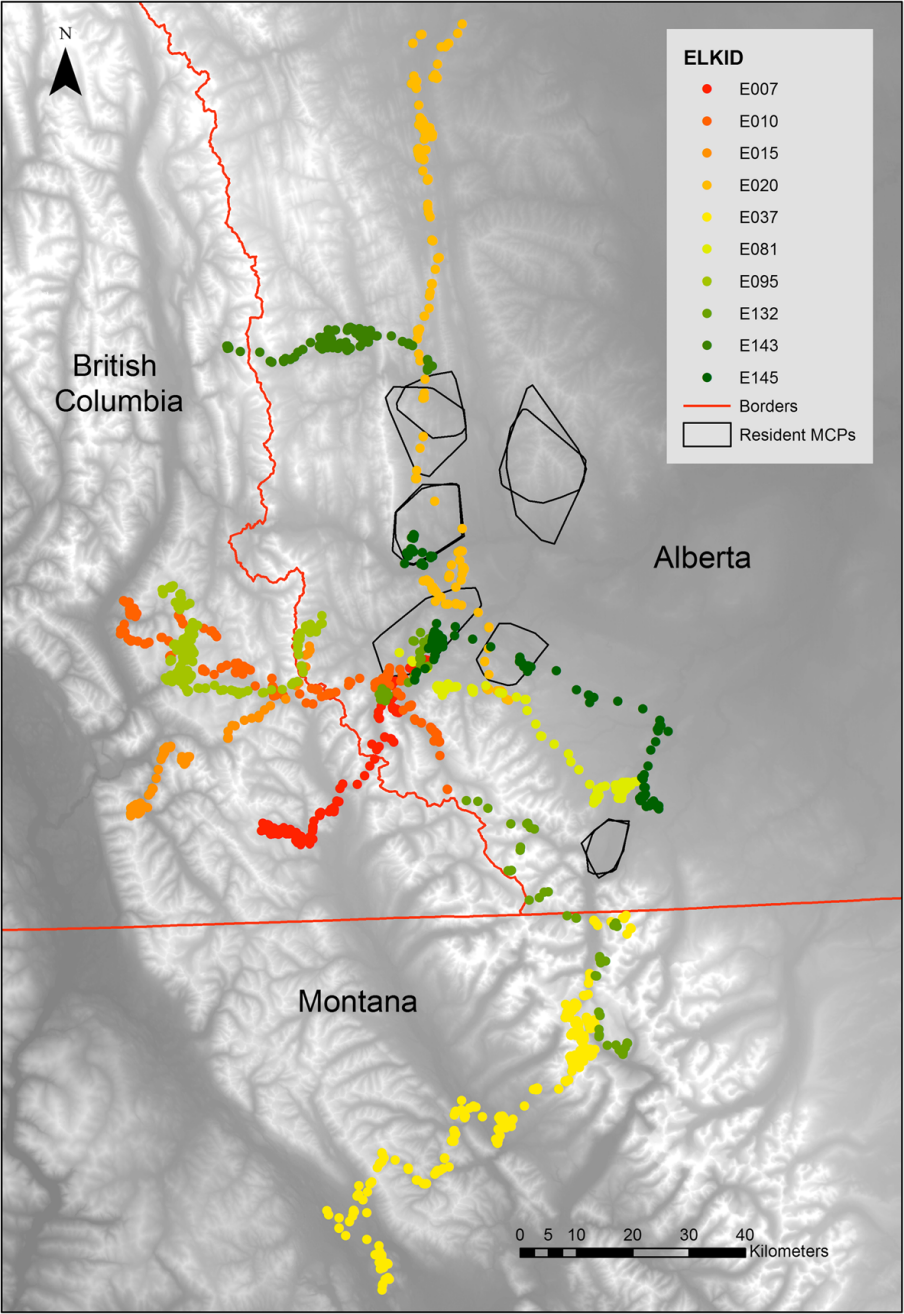
**The study area and telemetry data overview.** Map of the study area, located in Alberta and British Columbia, Canada and Montana, USA. Telemetry data was collected between 2007 and 2011. Locations from dispersing elk, during their dispersal period, are shown as points (each colour corresponds to an individual elk) on a digital elevation model of the area (lighter areas representing higher elevation). Minimum convex polygons for the full year of data for each matched resident are also shown in black.

Dispersal events ranged in length from 29.16 km to 98.01 km (straight line distance from first to last location, actual distance travelled greater) and lasted from 12 to 47 days (M = 25.9 days), taking place between 18-May and 04-August, with the majority of movement occurring in June and July. Dispersal routes traversed a range of landscapes, with some animals travelling from Alberta into British Columbia or Montana (Figure 1). Exploratory movements were similar to dispersal movements in terms of timing and distance travelled but the animals returned to their starting locality. These movements occurred on a timescale of weeks, inconsistent with migration, and we consider them as explorations (Additional file 1: Figure S1). All males were captured at approximately age 1.5 and therefore were dispersing when just over 2 years old.

**Figure 2:**
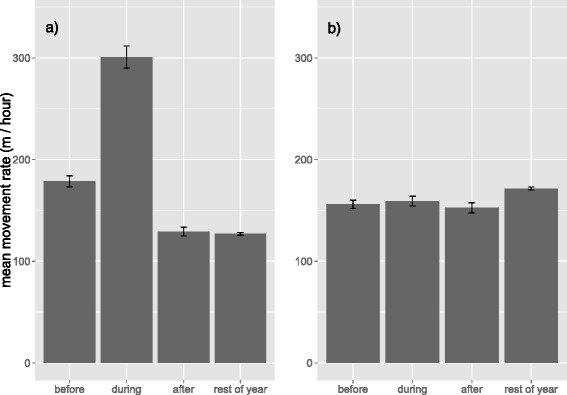
**Movement rate of dispersers and residents.** Movement rates (m hr^-1^) before, during and after the dispersal event and throughout the rest of the year for **a)** dispersers and **b)** residents The mean movement rate in each period is shown with error bars representing ±1 SE. For dispersers, movement rate in the during-dispersal time period was significantly greater than in all other time periods and the before-dispersal period was also greater than the after-dispersal period, which was not different from the rest of the year. For residents, there was no significant difference in mean movement rate before, during and after the dispersal period, and in all these periods they moved slower than during the rest of the year.

For dispersers, according to the predictions of linear mixed effects analysis, movement rate (m hr^-1^) recorded during different time periods (Figure 2) had the following pattern: during dispersal (β = 173.9; 95% CI 162.4, 185.3) > > before dispersal (β = 57.5; 95% CI 46.5, 68.4) > > after dispersal (β = 4.0, 95% CI -7.28, 15.3). Movement rate after dispersal did not differ from that recorded throughout the rest of the year (reference category, β = 0). Residents meanwhile did not show significant differences in movement rate between the periods during (β = -10.5, 95% CI -20.7, -0.48; Figure 2), before (β = -14.3; 95% CI -24.5, -4.1) and after (β = -20.6; 95% CI -31.5, -9.7), although movement rate in all was lower compared to that recorded during the rest of the year (reference category, β = 0).

**Figure 3:**
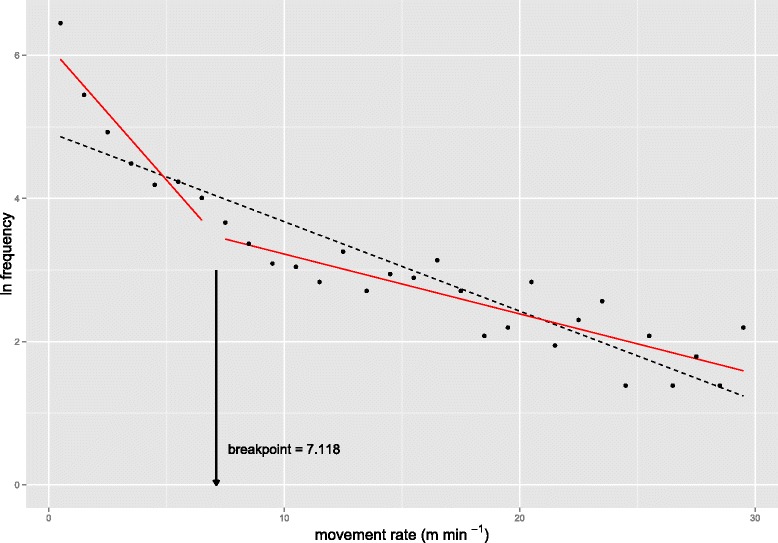
**Segmented regression of movement rate.** The log_e_ frequency distribution of movement rates for all dispersers combined, during their dispersal period. Movement rates were binned and the log_e_ frequency in each bin was plotted, *sensu* Johnson et al. 2002 [30]. A segmented linear regression model was fit (red lines, r^2^ = 0.93) for which a breakpoint of 7.118 m min^-1^ was identified using the R package *segmented*. A null model linear regression model is also shown for comparison (black, dashed line, r^2^ = 0.82).

Movement data during the dispersal period were further divided into two parts using segmented regression, representing two distinct movement behaviours [30]. The breakpoint was identified as a movement rate of 7.12 m min^-1^ (SE = 1.04) which corresponds to a step length of 854 m in 2 hours (Figure 3).

**Figure 4:**
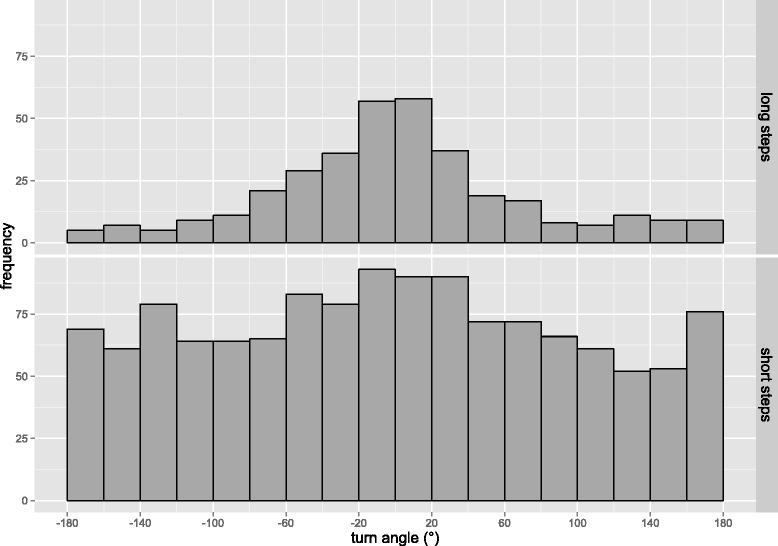
**Turning angle distributions for short and long steps during dispersal.** Turning angle distributions combined for the 10 dispersers during their dispersal period are shown. The upper graph shows the distribution of turning angles for steps longer than 854 m (*n* = 355 steps) and the lower graph shows the distribution of turning angles for steps shorter than 854 m (*n* = 1,292 steps). This boundary was derived from the segmented regression breakpoint in Figure 3.

This resulted in two subsets of data – one of steps under 854 m, representing shorter steps during foraging or resting (n = 1,292 steps), and the other of steps over 854 m, representing longer movement steps (n = 355 steps). Turning-angle distribution was distinct between the groups, with longer steps having smaller turning angles and therefore being more directional (Figure 4). Turning angles associated with short steps also are burdened with greater sampling error [31].

### Fine-scale habitat selection

**Figure 5:**
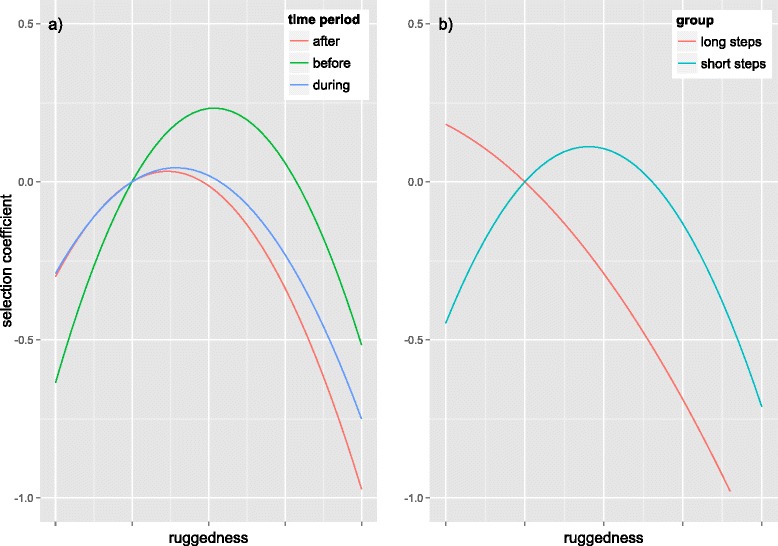
**The response of dispersers to ruggedness.** The relationship between the selection coefficients (beta values) for ruggedness. In **a)** dispersers are shown for the before, during and after periods of dispersal. Avoidance of ruggedness is stronger during and after dispersal compared to before dispersal. In **b)** the long step and short step groups, as derived from segmented regression (Figure 3), are shown for the dispersers during their dispersal period. Avoidance of ruggedness appears stronger for long steps than for short steps.

For dispersers the relationship between step length and terrain ruggedness was strongly non-linear with selection for intermediate values of ruggedness during all time periods (Table 2). They avoided more rugged terrain in all time periods, but this avoidance was weaker before dispersal (Figure 5a). Dispersers also had a weak attraction to areas with high Normalized Difference Vegetation Index (NDVI) values and to some extent avoided areas with high canopy cover, strongly before dispersal and weakly during and after dispersal, with no evidence for a non-linear effect. Distance to roads had no or a weak effect in all periods examined.

**Table 2:** **SSF model parameter estimates (fine-scale)**

	**Ruggedness**	**Ruggedness ^2**	**NDVI**	**Distance roads**	**Canopy cover**	**Canopy cover ^2**
	**Beta (se)**	**Beta (se)**	**Beta (se)**	**Beta (se)**	**Beta (se)**	**Beta (se)**
Dispersers before	**0.434** (0.100)	**- 0.202** (0.052)	0.623 (0.416)	0.457 (0.242)	**- 0.238** (0.108)	- 0.185 (0.172)
Dispersers during	0.155 (0.097)	**- 0.135** (0.044)	0.288 (0.175)	0.113 (0.158)	- 0.113 (0.082)	- 0.053 (0.068)
Dispersers after	0.144 (0.106)	**- 0.156** (0.035)	0.860 (0.443)	0.354 (0.203)	- 0.186 (0.139)	- 0.002 (0.129)
Residents before	- 0.112 (0.125)	**- 0.281** (0.081)	**1.031** (0.345)	0.202 (0.153)	0.132 (0.092)	**- 0.291** (0.078)
Residents during	- 0.087 (0.146)	- 0.101 (0.085)	**0.996** (0.309)	0.177 (0.169)	**0.238** (0.072)	**- 0.216** (0.065)
Residents after	0.111 (0.075)	- 0.103 (0.085)	**0.598** (0.209)	0.047 (0.188)	**0.229** (0.095)	**- 0.263** (0.086)
Disp. during long-steps	- 0.236 (0.151)	- 0.054 (0.054)	0.066 (0.278)	- 0.114 (0.298)	- 0.121 (0.108)	- 0.029 (0.119)
Disp. during short-steps	**0.276** (0.117)	**- 0.171** (0.057)	0.385 (0.227)	0.211 (0.136)	- 0.073 (0.102)	- 0.099 (0.085)

Beta values with standard errors in brackets are shown, from each of the SSF models. Beta values greater than 2 SE from 0 are shown in bold.

During the dispersal period, the shorter steps, as identified by segmented regression, were still characterised by strong avoidance of more rugged terrain and selection for intermediate values. Longer steps showed a linear avoidance of ruggedness (Figure 5b), but this model was not highly predictive. The short-step group of steps were more likely to end in resource units with higher NDVI values while the long-step group had no effect of NDVI (Table 2).

For residents there was also a non-linear relationship between step length and terrain ruggedness but this effect was only strong in one time period. There was in general a weak avoidance of more rugged terrain, but in contrast to dispersers there was a strong attraction to areas with high NDVI values in all time periods. There was also a non-linear relationship with canopy cover in all time periods with residents selecting intermediate values while strongly avoiding areas with high canopy cover (Table 2).

All models, except for the long-steps model, were found to be useful predictors of habitat selection, with the mean observed r_S_ of the models greater than 0.65, and the distribution of r_S_ higher than expected by chance alone (Additional file 2: Table S1).

### Broad-scale habitat selection

Using correlated random walks with turning angle and step-lengths from the distribution of observed during-dispersal long steps, we compared each dispersal pathway to random paths. For 7 of the 10 animals there was an avoidance of rugged terrain at this broad scale but overall this was not significantly different from zero (mean β = -0.66, Wilcoxon signed rank test, *P* = 0.28). However, for NDVI there was a more consistent attraction to higher values (mean β = 0.99, Wilcoxon signed rank test, *P* = 0.03) with 8 of 10 animals showing selection. We found no significant pattern with canopy cover nor distance to roads (Wilcoxon signed rank test, mean β = -0.03, *P* = 0.85; mean β = 0.25 *P* = 0.28 respectively).

## Discussion

### Dispersal and movement parameters

We observed a number of substantial long-distance dispersal events, all but one of which was made by males. Of the 38 males tracked for the required time period 39% had a dispersal period (24% completed) which is comparable to previous estimates of 27% for herds in Colorado [25] and 40% in a Montana herd [32]. This pattern of male-biased dispersal is expected for a polygynous mammal species, because males have highly variable reproductive success [19]. Our estimate is, if anything, likely to be an underestimate because many males (28% of those tracked) were killed before we were able to gather enough data to confirm if they were dispersers or migrants. Given the lack of male residents and female dispersers, we cannot assess the effect of sex on our models. However, we had a large sample size and age range of females and it is clear that dispersal is almost totally male-dominated and therefore the characteristics of dispersal behaviour appear inextricably linked to males.

Movement rate for dispersers during the dispersal periods was greater than movements before or after. This shows that the extracted dispersal period is indeed distinct in terms of movement rate. Dispersing animals move faster and further and maintain a persistent direction. There was a significant difference between the before dispersal movement rate in comparison to the rest of the year, possibly representing a period of restlessness in the pre-dispersal phase. In the period after dispersal movement rate reduced, during which time there was likely to be a slow formation of a new home-range. Within their home ranges animals are likely to switch between long directional movements between patches and periods of short non-directional movements within a patch [27]. This is probably based on spatial memory of resources within their home-range [33]. However, seasonality also may affect movement rate of animals and this was not explicitly examined, although resident and dispersing animals were paired in time to account for seasonal effects. Resident animals had similar movement rates in all time periods, while dispersers did not.

### Fine-scale habitat selection

Steps taken by dispersing elk showed a non-linear relationship with terrain ruggedness in all time periods examined, selecting intermediate values of ruggedness, while avoiding the most rugged terrain. Avoidance of rugged terrain has been observed for other mammals [13,34] and presumably reduces energy expenditure and facilitates ease of movement [35]. Unexpectedly, the response by dispersing elk to terrain ruggedness was similar both during and after dispersal. This may be because these individuals were experiencing novel environments and minimising energy expenditure while exploring and forming a new home-range after dispersal. We also split the during-dispersal period into two movement behaviours. A variety of methods have been used to infer different movement behaviours from step-length data, providing insight into movement decisions [27,30,36]. We found that when only looking at long movement steps, in comparison to short steps during foraging or resting, the avoidance of ruggedness was linear for longer steps. More avoidance of rugged terrain during longer steps might be expected given that only when there is considerable movement is the energy saved by avoiding rugged terrain likely to be substantial, although steps may be shorter in rugged terrain due to the difficulty of movement. However, the long-steps model was not validated, showing that habitat selection for these long movement steps may be somewhat unpredictable.

In contrast, resident elk had inconsistent responses to ruggedness, although did still avoid more rugged terrain. However unlike dispersers, they showed strong and highly consistent selection for areas with higher NDVI values. High values of NDVI indicate green herbaceous phytomass or forage quantity [37] suggesting that residents are selecting areas with high forage availability the majority of the time. This is consistent with previous studies showing that foraging dominates summer elk activity [36,38]. During dispersal we expected that short steps would show a strong attraction to high NDVI values in comparison to long steps, because they should be associated with foraging when moving less rapidly. In fact this pattern was weak, and it may be that dispersers foraged wherever possible, rather than being highly selective, or that the pattern of foraging might be obscured with steps during resting behaviour, all of which fall within the same group. Differentiating resting and foraging steps is difficult given the imprecision inherent in GPS data [36]. Residents also showed avoidance of areas with high canopy cover and selected movement toward localities with lower canopy cover, consistent with elk use of forest cover for protection while using open areas for foraging [39].

### Broad-scale habitat selection

While habitat selection at fine scale examines behavioural decisions over the course of hours, we also used broad-scale analyses to examine characteristics of the dispersal pathway as a whole. At this larger scale we found that there was no clear overall avoidance of rugged terrain, which was surprising given the strength of the relationship at fine scale. This suggests that although dispersing animals appear to select habitats for ease of movement at fine scale during movement, at a larger scale they do not necessarily select their overall route in this way. This could be because of imperfect knowledge of their environment – dispersers are travelling through areas that they have never encountered before, but perhaps also because they select the direction of dispersal based on a broad assessment of habitat and forage quality and along that route select habitats for ease of movement at the fine scale. This is supported by the overall positive relationship of movement toward resource units with high NDVI values.

## Conclusions

In one of the first examples of its kind we were able to track dispersing elk using high fix-rate GPS satellite radiotelemetry throughout the entirety of their dispersal periods, providing detailed information on the distance and direction travelled and the likelihood of dispersal. By analysing habitat selection with these data we find evidence to suggest that the most-supported step selection functions during dispersal are not the same as those selected for home-ranging behaviour. Where possible it would be beneficial to estimate and account for the differences when using cost-distance modelling. If managers are to implement initiatives to promote habitat connectivity, such as corridors, it would be ideal to prioritise data collection from individuals that are most likely to disperse. This serves a double purpose. One, it ensures that the data obtained are as representative as possible for characterising dispersal movement, the primary purpose for habitat corridors. Second, dispersers cover much larger distances during their travel than other animals, making them a cost-effective way to get the maximum amount of data about habitat selection throughout a landscape. For ungulates, it would be most useful to track young males, especially during spring and early summer, because these are the animals most likely to disperse.

Overall we believe that the processes of dispersal amongst large mammals warrant further investigation and modern GPS radiotelemetry provides an excellent tool in this endeavour. We also find step selection functions to be a powerful statistical tool for analysing habitat selection during dispersal.

## Methods

### Study area

The study took place in southwest Alberta and extended into northwest Montana and southeast British Columbia (Figure 1). The majority of the area within Alberta is provincial forest reserve and on the eastern boundary of our study area there is mixed livestock ranching and cropland. This boundary is a transition zone from grassland into the Rocky Mountains and several different elk populations are present. Natural predators of elk in the area are wolf (*Canis lupus*), cougar (*Puma concolor*), and grizzly bear (*Ursus arctos*) [40]. There is considerable human presence in the study area, including industrial activities such as forestry and natural gas extraction, as well as recreational activities, especially during summer and the autumn hunting season [41].

### Elk data

A total of 158 elk (105 female, 53 male) were captured on winter ranges between 2007 and 2011 using helicopter net-gunning. Each was fitted with a GPS-radiotelemetry collar (either ARGOS GPS for males or Lotek GPS 4400 for females, Lotek Wireless Inc., Ontario, Canada) and all units were programmed to obtain locations every 2 hrs. Satellite transmitted data from GPS collars fitted with Argos communication devices were received weekly via email, while other data were downloaded remotely in the field. Radiocollars from elk that died were located and re-fitted to new animals. All males were approximately 1.5 yrs old at time of capture and females varied between 1.5 yrs and 19 yrs old. A vestigial canine was removed during capture and used to determine age by cementum analysis (Matson’s Laboratory, MT, USA). Locations were screened following the method of Lewis et al. 2007 [42] but a few large measurement errors remained. These outliers were easily identified as locations which were an unreasonable distance from the previous and next location (10s of km round trips in 4 hrs) and were removed. Data sets also were trimmed at beginning and end to remove data where an elk’s behaviour might have been influenced by capture or where the elk had died or where the collar failed to function. Fix rate for the 20 animals included in the analysis was 81.7% for Lotek 4400 (females) and 66.2% for ARGOS GPS (males).

### Distinguishing movement types

Only data from individuals with relocations from at least 1^st^ April to 31^st^ October were included and of the 158 radio-collared animals, 26 (15 males, 11 females) were excluded. This was to ensure that the entirety of the migration or dispersal period was covered. To distinguish between different movement behaviours we used the measurement Net-Squared Displacement (NSD) [28]. This is a time-dependent statistic that measures the straight-line distance between a starting location and subsequent locations in a movement path of a given individual. We used the method of Bunnefeld et al. [43] to classify each animal as migrant or resident and to identify dispersal events. Graphs of NSD were then inspected visually to identify the type of movement, similar to Mysterud et al. 2011 [44]. After a successful dispersal animals will settle into a new home range and become either migrants or residents and in some cases there were available data to classify these animals as migrant or resident, as well as their dispersal event. Those without long enough periods of data following dispersal were classified as other/not classified, along with a number of animals with atypical movement patterns. Several males had NSD graphs suggestive of dispersal, but were killed by hunters during the autumn hunting season (September to end of November for rifle hunting [45]), making it difficult to confirm if they were truly dispersers, or migratory. A number of individuals also showed an ‘exploratory movement’ (Additional file 1: Figure S1). These movements were similar to dispersal movements, occurring within the same timeframe before migration, rapidly travelling a long distance, but then returning to or close to their original range within a short period of time. These appear to be exploration events, and therefore were included as dispersers in our analyses, although we note that our results were not notably altered by inclusion of these individuals. Our categorisation resulted in a group of elk-years with clearly distinguishable movement patterns (Additional file 1: Figures S1, S2). We calculated NSD and plotted the associated graphs using the *adehabitat* package version 1.8 [46] in version 2.15 of R [47].

### Data selection and movement parameters

To investigate habitat selection by dispersers we selected individuals that had a dispersal period (*n* = 10, 7 of which completed dispersal, 3 of which had exploratory movements) and for which there were resident individuals with data spanning the same timeframe (*n* = 10) available for comparison (*n* = 20 individuals, 57,637 telemetry relocations in total, locations shown in Figure 1). Residents were used for comparison because they have a simple movement pattern in which they stay within the same home range throughout the year. We did not investigate migrants because migration in this population is typically characterised by frequent stop-overs while following spring green-up towards summer ranges, rather than a single directed movement period [48]. This makes it difficult to compare the movement period of a migration directly with that of a dispersal period. We selected the telemetry points that occurred during the dispersal movement event itself, to the nearest day. This was done using NSD graphs, with the start of dispersal identified by a steep increase in the value of NSD and the end of dispersal identified by NSD plateauing as the animal settles into a new home range (Additional file 1: Figure S1). If there was a short movement followed by a period of a month or more of stationary behaviour, before the main dispersal event, this early movement was not considered to be part of the dispersal event itself.

Also, we selected telemetry locations in the period of 26 days (corresponding to the average duration of a dispersal event) immediately preceding and after the dispersal event. This gave us a group of data points from dispersers before they had undertaken their dispersal and after they had completed it. These same time periods also were analysed for matched residents even though there was no dispersal among these individuals.

Movement rate (m hr^-1^) was calculated by dividing the step length between two successfully obtained locations by the time elapsed between those locations. We used a mixed effects model with individual elk as a random factor to assess the effect of the time period (before, during, after dispersal, and the rest of the year) on movement rate. Movement rate throughout the rest of the year was set as reference category in the mixed model. A separate model was used for dispersers and for residents, using the R package lme4 [49]. In both models, statistically significant differences (*α* = 0.05) between movement rates recorded during different time periods were assessed by checking the overlap of 95% Confidence Intervals (CIs), assuming movement rates to be statistically different (*P* < 0.05) if related 95% CIs didn’t overlap.

### Landscape variables

We identified a number of environmental covariates that are known to influence elk movement behaviour [45,50]. Each covariate was imported into a GIS system and values for each were then attached to the points in the telemetry dataset using ESRI ArcGIS 10.1 (ESRI Inc., Redlands, CA) and GME (Geospatial Modelling Environment, http://www.spatialecology.com/gme/).

We used a 30-m resolution Digital Elevation Model (DEM) and the Spatial Analyst extension in ESRI ArcGIS 10.1 to produce a terrain ruggedness variable (30-m resolution), which quantifies topographic heterogeneity [51]. This is a measurement of the average elevation difference between a point on a digital elevation model grid and the surrounding cells. We included both a linear and a squared term given an expected non-linear relationship to ruggedness.

As a proxy for forage quality we used monthly Normalized Difference Vegetation Index (NDVI) measurements at 250-m resolution (1 layer per month, MODIS Science Team, http://modis.gsfc.nasa.gov/). Chlorophyll strongly absorbs visible light but strongly reflects near-infrared light allowing the use of remote sensing to measure and compare the intensity of light emitted in these wavelengths, thereby enabling photosynthetic capacity of vegetation to be quantified [37]. This measurement is widely used in ecological studies, being highly correlated with green herbaceous phytomass [52]. Although high NDVI values from conifer forested areas may not necessarily represent the ground-level forage availability, ground estimates of herbaceous forage biomass for elk correlate with satellite derived NDVI values in tree-covered vegetation types [22].

A measure of distance to roads was used, derived from road maps of the study area including both paved and gravel roads (Governments of AB & BC: National Topographic Database 1:50,000; U.S. Census Bureau Tiger/Line files, 2000). Finally we used a percent canopy cover surface at 30 m resolution (U.S. National Land Cover Database, Governments of Alberta/British Columbia). Again, we included both a linear and a squared term given an expected non-linear relationship to canopy cover.

### Modelling fine-scale habitat selection using step selection functions

The straight-line segments linking successive animal locations, taken at regular intervals, are defined as steps [53]. We calculated step length in metres and turning angle (angle between previous and next location) using GME with ARCMAP v. 10.1 (ESRI Inc., Redlands, CA). In analyses involving step length, we used only steps where the time interval was within 10 minutes of the typical two-hour interval. Steps with intervals much shorter or longer were removed to ensure fair comparison of step length.

Step selection functions (SSF) are used for incorporating movement into habitat selection analysis, providing a more fine-scale and mechanistic movement model than the original Resource Selection Function (RSF) [54]. In an SSF each observed step is compared with a number of random steps that have the same starting point. Random step lengths and turning angles are drawn from distributions taken from observed data, allowing comparison of the observed step to a sample of those that could have been taken in the local environment. Here we analyse the endpoints of observed and random steps, similar to a conditional RSF but with controls drawn from a domain most likely to represent those truly available given observed movement patterns [29].

Step lengths and turn angles for the random steps were drawn independently from distributions of the observed values, which was reasonable because of low observed correlation between step length and turning angle [29]. Observations of turn angle and step length were placed into 10° bins and 50 m bins respectively. A unique distribution for each individual was created by calculating a probability distribution using values from all other animals excluding the individual itself, thereby avoiding problems of circularity [54]. These distributions were then used to create 10 random steps per real step using GME with ARCMAP v. 10.1.

### SSF analysis

We developed separate models for dispersers in the time periods before, during and after dispersal as well as models for matched residents during these same time periods. We split the during-dispersal subset of data using a segmented regression or ‘broken-stick’ model to define two types of movement behaviour (shorter steps during resting and foraging and less-frequent long movement steps) [30]. This was carried out using the R package *segmented* [55]. Two further models were then fitted to each of these subsets. For ease of comparison between time periods we used the same explanatory variable structure for all models, *sensu* Muhly et al. 2010 [50]. To allow comparison between variables we scaled each variable by subtracting the mean and dividing by the standard deviation of the input variable.

For all models we used a two-stage modelling approach, fitting models first to individuals and then averaging parameters across individuals to estimate population-level selection parameters [56]. Before including variables we evaluated them in terms of collinearity and multicollinearity using Pearson correlation coefficients (r) and variance inflation factors (all r < 0.3 and all VIF < 2) as well as for biological meaningfulness. We used conditional logistic regression to estimate SSFs assuming an exponential selection function of the form:

$$ \widehat{w}\left(\mathbf{x}\right)= \exp \left({\beta}_1{x}_1+{\beta}_2{x}_2+{\beta}_3{x}_3+\dots +{\beta}_p{x}_p\right) $$

where β_1_ to β_p_ are coefficients estimated by conditional logistic regression, which are associated with a vector **x**, of environmental variables *x*_1_ to *x*_*p*_ respectively. The higher the value of $\widehat{w}\left(\mathbf{x}\right)$ the more likely that step will be chosen by an animal [57].

The analysis was carried out using version 1.2 of the R package *TwoStepCLogit* [58]. To correctly analyse data in which there are multiple controls per case it is necessary to use conditional logistic regression. However, it is difficult to take into account cluster-level variation (i.e., random variation among elk) using this method, and this is important to address given that resource selection can vary considerably between individuals within a population and large differences in individual behaviour have been observed amongst elk [45,59]. To account for variation among individuals the *TwoStepCLogit* package was used to first fit fixed-effects regression models to each individual elk and then combine those estimates using restricted maximum likelihood (REML) estimation [58]. This method gives stable and consistent estimations of the parameters in mixed conditional logistic regression models when the number of strata is large, as it was in this dataset. By using this procedure we account for the large inter-individual variation observed amongst the elk. The two-stage approach also helps to account for correlation within individuals, common in habitat-selection studies (autocorrelation) [56]. Models were validated using *k*-fold cross validation [60].

### Habitat selection at the broad-scale using correlated random walk analysis of dispersers

We tested for the landscape characteristics defining real dispersal routes at a broad-scale by comparing each route to 10 random alternatives created using the *simple.crw* function in GME. Step length and turning angle distributions were specified from the observed distributions from each individual, using only the long movement steps, as defined by the segmented regression model. This ensured that random alternatives were of a comparable length to the actual dispersal route. We used the same landscape variables as in the previous analysis and compared the observed points during dispersal to the random points derived from the correlated random walks. We used a generalised linear model per individual with a binomial link function and averaged the resulting coefficients for a population estimate. We then performed one-sample Wilcoxon signed rank tests for each variable to determine if the beta averages were significantly different from zero.

## Additional files

Additional file 1:
**Net Squared Displacement graphs for individuals included in SSF analyses.** **Figure S1.** Net Squared Displacement (NSD) calculated for *n* = 10 dispersers (D), all of which are male. Of the dispersers, E007, E010 and E095 undergo exploratory movements in which they return to, or close to, previous ranges. Beside each individual is the NSD graph for the extracted dispersal period. **Figure S2.** Net Squared Displacement (NSD) calculated for *n* = 10 residents (R), all of which are female. Additional file 2: Table S1.
*k*-fold cross-validation results. Robustness of models was evaluated by *k*-fold cross validation for case-control design, following the method of Fortin et al. 2009 [60]. The SSFs were built using a random selection of 80% of the data strata and then used to predict the SSF scores for the remaining 20% of strata. The observed location was then ranked against the random locations, for each stratum, and ranks were tallied. Spearman rank correlations (r_S_) were carried out with the bin’s ranking and associated frequency. The procedure was repeated 100 times and the mean and range of r_S_ are shown. The mean and expected range of r_S_ are also shown, calculated by ranking one random location against the other random locations, per strata, tallying ranks and using Spearman rank correlations (r_S_). Again this was repeated 100 times.
