# Supplementary material for: Habitat selection during ungulate dispersal and exploratory movement at broad and fine scale with implications for conservation management
Source: Mov Ecol. 2014 Jul 26;2:15. doi: 10.1186/s40462-014-0015-4 (PMC4855897; doi:10.1186/s40462-014-0015-4)

Additional File Figure S1: Net Squared Displacement (NSD) calculated for *n* = 10 dispersers (D), all of which are male. Of the dispersers, E007, E010 and E095 undergo exploratory movements in which they return to, or close to, previous ranges. Beside each individual is the NSD graph for the extracted dispersal period.

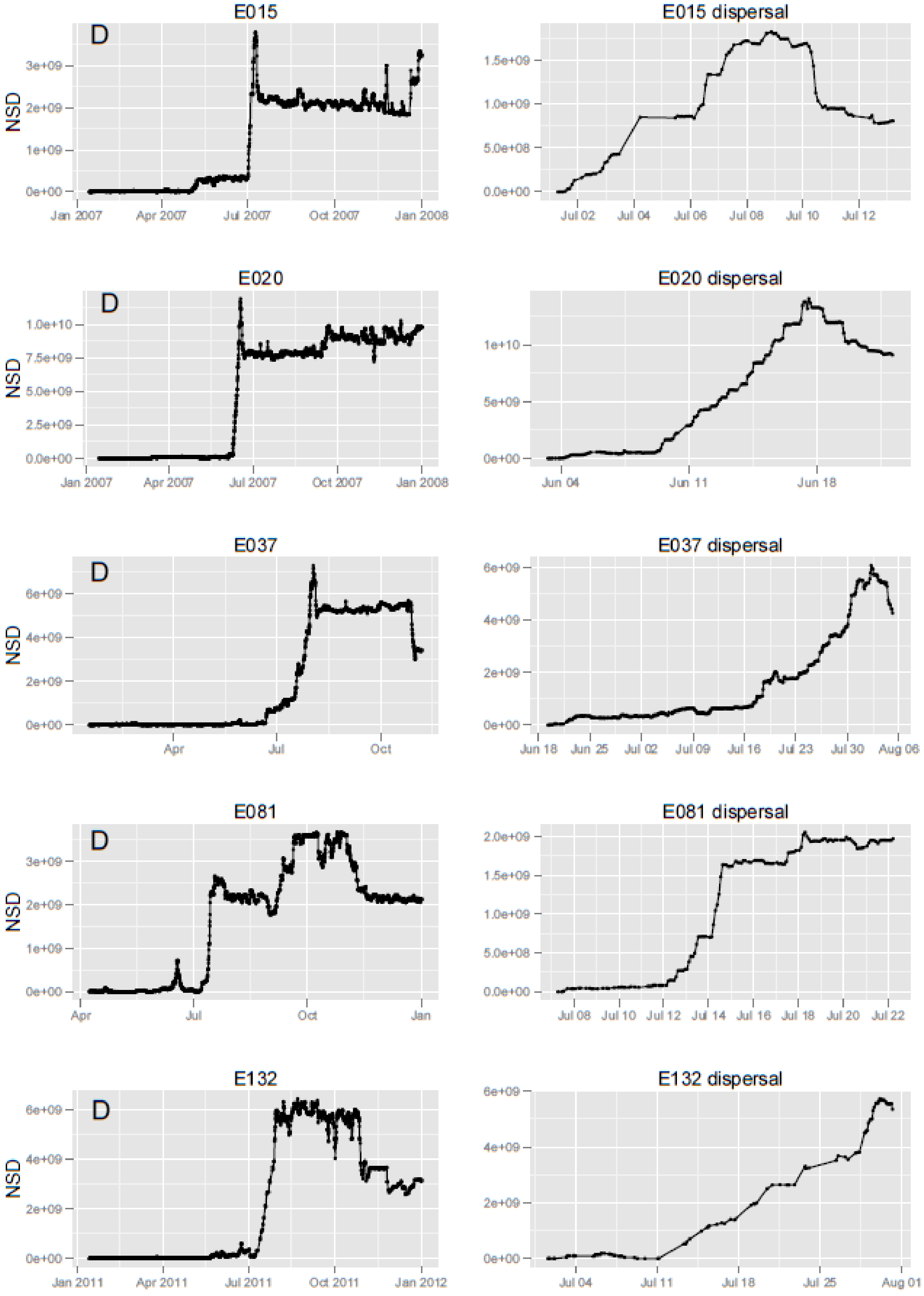

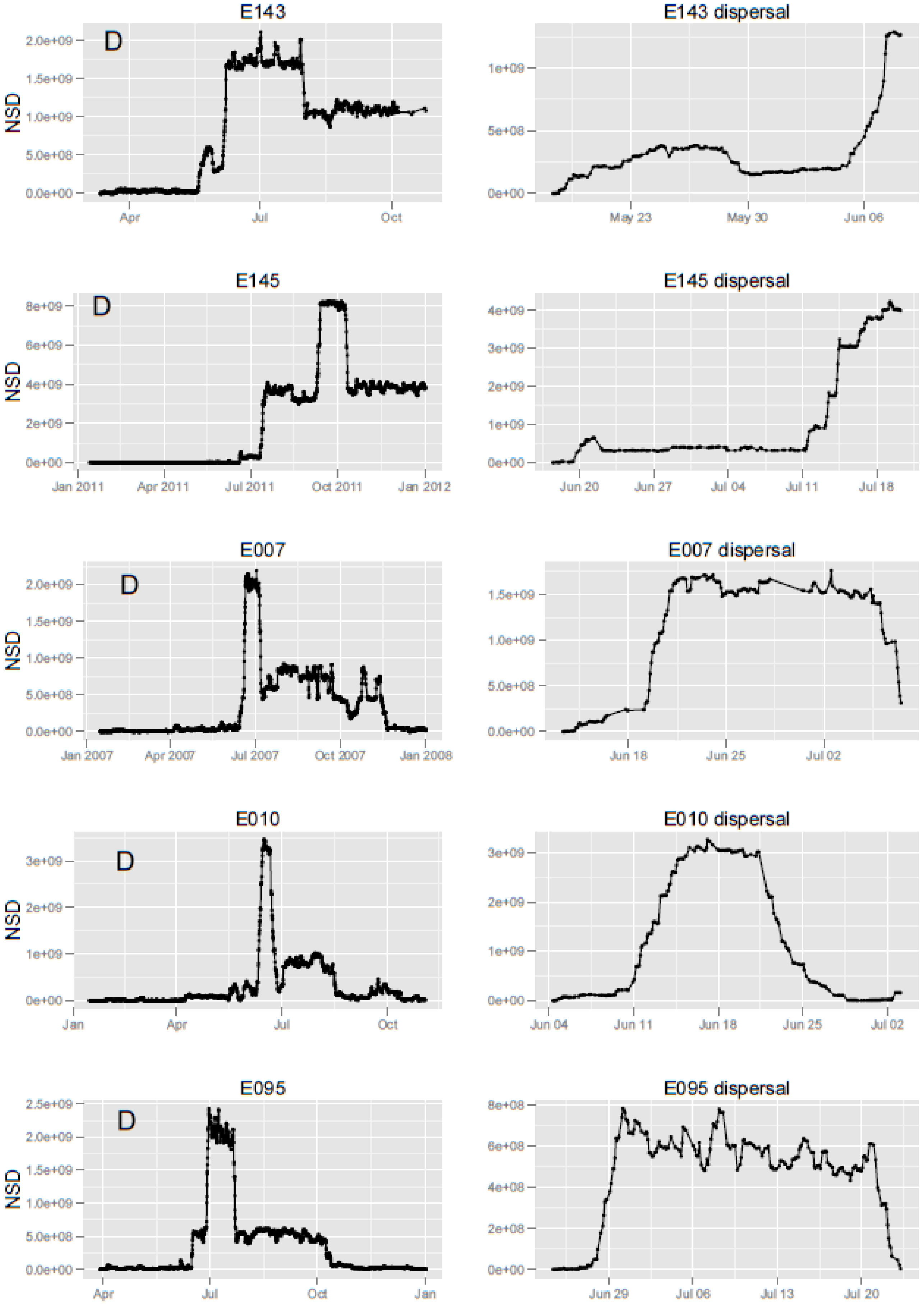
E143
D
NSD
2.0e+09
1.5e+09
1.0e+09
5.0e+08
0.0e+00
Apr
Jul
Oct
E143 dispersal
1e+09
5e+08
0e+00
May 23
May 30
Jun 06
E145
D
NSD
8e+09
6e+09
4e+09
2e+09
0e+00
Jan 2011
Apr 2011
Jul 2011
Oct 2011
Jan 2012
E145 dispersal
4e+09
3e+09
2e+09
1e+09
0e+00
Jun 20
Jun 27
Jul 04
Jul 11
Jul 18
E007
D
NSD
2.0e+09
1.5e+09
1.0e+09
5.0e+08
0.0e+00
Jan 2007
Apr 2007
Jul 2007
Oct 2007
Jan 2008
E007 dispersal
1.5e+09
1.0e+09
5.0e+08
0.0e+00
Jun 18
Jun 25
Jul 02
E010
D
NSD
3e+09
2e+09
1e+09
0e+00
Jan
Apr
Jul
Oct
E010 dispersal
3e+09
2e+09
1e+09
0e+00
Jun 04
Jun 11
Jun 18
Jun 25
Jul 02
E095
D
NSD
2.5e+09
2.0e+09
1.5e+09
1.0e+09
5.0e+08
0.0e+00
Apr
Jul
Oct
Jan
E095 dispersal
8e+08
6e+08
4e+08
2e+08
0e+00
Jun 29
Jul 06
Jul 13
Jul 20

Additional File Figure S2: Net Squared Displacement (NSD) calculated for *n* = 10 residents (R), all of which are female.

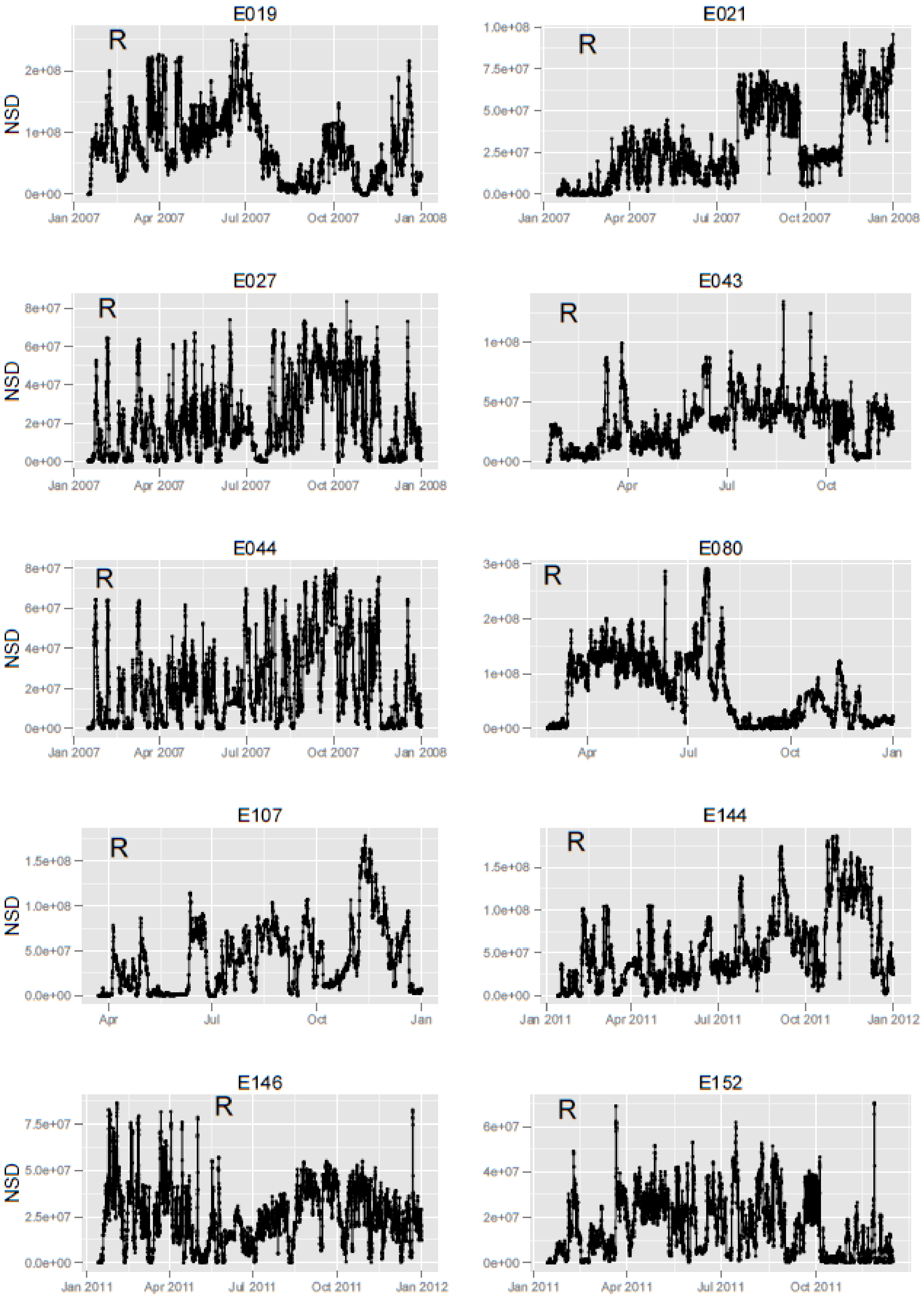

Supplement: Additional file 1: — Net Squared Displacement graphs for individuals included in SSF analyses. Figure S1. Net Squared Displacement (NSD) calculated for n = 10 dispersers (D), all of which are male. Of the dispersers, E007, E010 and E095 undergo exploratory movements in which they return to, or close to, previous ranges. Beside each individual is the NSD graph for the extracted dispersal period. Figure S2. Net Squared Displacement (NSD) calculated for n = 10 residents (R), all of which are female. [file 40462_2014_15_MOESM1_ESM.pdf]
