# Supplementary material for: Habitat selection during ungulate dispersal and exploratory movement at broad and fine scale with implications for conservation management
Source: Mov Ecol. 2014 Jul 26;2:15. doi: 10.1186/s40462-014-0015-4 (PMC4855897; doi:10.1186/s40462-014-0015-4)
Supplement: Additional file 2: Table S1. — k-fold cross-validation results. Robustness of models was evaluated by k-fold cross validation for case-control design, following the method of Fortin et al. 2009 [60]. The SSFs were built using a random selection of 80% of the data strata and then used to predict the SSF scores for the remaining 20% of strata. The observed location was then ranked against the random locations, for each stratum, and ranks were tallied. Spearman rank correlations (rS) were carried out with the bin’s ranking and associated frequency. The procedure was repeated 100 times and the mean and range of rS are shown. The mean and expected range of rS are also shown, calculated by ranking one random location against the other random locations, per strata, tallying ranks and using Spearman rank correlations (rS). Again this was repeated 100 times. [file 40462_2014_15_MOESM2_ESM.pdf]

Additional File Table S1: K-fold cross-validation results. Robustness of models was evaluated by k-fold cross validation for case-control design, following the method of Fortin et al. 2009 [1]. The SSFs were built using a random selection of 80% of the data strata and then used to predict the SSF scores for the remaining 20% of strata. The observed location was then ranked against the random locations, for each stratum, and ranks were tallied. Spearman rank correlations (r_S) were carried out with the bin's ranking and associated frequency. The procedure was repeated 100 times and the mean and range of r_S are shown. The mean and expected range of r_S are also shown, calculated by ranking one random location against the other random locations, per strata, tallying ranks and using Spearman rank correlations (r_S). Again this was repeated 100 times.

**model**	**obs. r_S value (range)**	**random obs. value (range)**
dispersers before	0.774 (0.460 – 0.980)	-0.018 (-0.767 – 0.753)
dispersers during	0.723 (0.192 – 0.980)	0.004 (-0.821 – 0.721)
dispersers after	0.678 (0.182 – 0.925)	0.010 (-0.614 – 0.663)
residents before	0.675 (0.128 – 0.945)	0.015 (-0.709 – 0.693)
residents during	0.740 (0.343 – 0.955)	0.046 (-0.879 – 0.758)
residents after	0.698 (0.110 – 0.943)	-0.017 (-0.718 – 0.745)
disp. during long steps	0.064 (-0.446 – 0.685)	0.051 (-0.774 – 0.800)
disp. during short steps	0.676 (0.270 – 0.973)	-0.007 (-0.693 – 0.693)

References

1. Fortin D, Fortin ME, Beyer HL, Duchesne T, Courant S, Dancose K: **Group-size-mediated habitat selection and group fusion-fission dynamics of bison under predation risk.** *Ecology* 2009, **90:**2480-2490.
